# Working with troubles and failures in conversation between humans and robots: workshop report

**DOI:** 10.3389/frobt.2023.1202306

**Published:** 2023-12-01

**Authors:** Frank Förster, Marta Romeo, Patrick Holthaus, Luke J. Wood, Christian Dondrup, Joel E. Fischer, Farhana Ferdousi Liza, Sara Kaszuba, Julian Hough, Birthe Nesset, Daniel Hernández García, Dimosthenis Kontogiorgos, Jennifer Williams, Elif Ecem Özkan, Pepita Barnard, Gustavo Berumen, Dominic Price, Sue Cobb, Martina Wiltschko, Lucien Tisserand, Martin Porcheron, Manuel Giuliani, Gabriel Skantze, Patrick G. T. Healey, Ioannis Papaioannou, Dimitra Gkatzia, Saul Albert, Guanyu Huang, Vladislav Maraev, Epaminondas Kapetanios

**Affiliations:** ^1^ Department of Computer Science, School of Physics, Engineering and Computer Science, University of Hertfordshire, Hatfield, United Kingdom; ^2^ Department of Computer Science, The University of Manchester, Manchester, United Kingdom; ^3^ School of Mathematical and Computer Sciences, Heriot-Watt University, Edinburgh, United Kingdom; ^4^ School of Computer Science, University of Nottingham, Nottingham, United Kingdom; ^5^ School of Computing Sciences, University of East Anglia, Norwich, United Kingdom; ^6^ Department of Computer, Control and Management Engineering “Antonio Ruberti”, Sapienza University of Rome, Rome, Italy; ^7^ School of Mathematics and Computer Science, Swansea University, Swansea, United Kingdom; ^8^ Department of Computer Science, Humboldt University of Berlin, Berlin, Germany; ^9^ Science of Intelligence, Research Cluster of Excellence, Berlin, Germany; ^10^ School of Electronics and Computer Science, University of Southampton, Southampton, United Kingdom; ^11^ School of Electronic Engineering and Computer Science, Queen Mary University of London, London, United Kingdom; ^12^ Institució Catalana de Recerca i Estudis Avançats, Universitat Pompeu Fabra, Barclona, Spain; ^13^ UMR 5191 ICAR, Centre National de la Recherche Scientifique, Labex ASLAN, ENS de Lyon, Lyon, France; ^14^ Computational Foundry, Faculty of Science and Engineering, Swansea University, Swansea, United Kingdom; ^15^ Department of Engineering, Design and Mathematics, University of the West of England, Bristol, United Kingdom; ^16^ Department of Speech Music and Hearing, KTH, Stockholm, Sweden; ^17^ Alana AI, London, United Kingdom; ^18^ School of Computing, Engineering and the Built Environment, Edinburgh Napier University, Edinburgh, United Kingdom; ^19^ School of Social Sciences and Humanities, Loughborough University, Loughborough, United Kingdom; ^20^ Department of Computer Science, The University of Sheffield, Sheffield, United Kingdom; ^21^ Department of Applied IT, Univeristy of Gothenburg, Göteborg, Sweden

**Keywords:** human-robot interaction, speech interfaces, dialogue systems, multi-modal interaction, communicative failure, repair

## Abstract

This paper summarizes the structure and findings from the first *Workshop on Troubles and Failures in Conversations between Humans and Robots*. The workshop was organized to bring together a small, interdisciplinary group of researchers working on miscommunication from two complementary perspectives. One group of technology-oriented researchers was made up of roboticists, Human-Robot Interaction (HRI) researchers and dialogue system experts. The second group involved experts from conversation analysis, cognitive science, and linguistics. Uniting both groups of researchers is the belief that communication failures between humans and machines need to be taken seriously and that a systematic analysis of such failures may open fruitful avenues in research beyond current practices to improve such systems, including both speech-centric and multimodal interfaces. This workshop represents a starting point for this endeavour. The aim of the workshop was threefold: Firstly, to establish an interdisciplinary network of researchers that share a common interest in investigating communicative failures with a particular view towards robotic speech interfaces; secondly, to gain a partial overview of the “failure landscape” as experienced by roboticists and HRI researchers; and thirdly, to determine the potential for creating a robotic benchmark scenario for testing future speech interfaces with respect to the identified failures. The present article summarizes both the “failure landscape” surveyed during the workshop as well as the outcomes of the attempt to define a benchmark scenario.

## 1 Introduction

Speech interfaces, user interfaces that allow interaction with technology through spoken commands or queries, are commonplace in many types of robots and robotic applications. Despite the progress in speech recognition and many other areas of natural language processing in recent years, failures of speech interfaces in robotic scenarios are numerous, especially in real-world situations (Porcheron et al., 2018; Fischer et al., 2019). In contrast to the common experience of failure of speech interfaces in robotics, the literature is positively skewed towards the success and good performance of these. While Marge et al. (2022) identified key scientific and engineering advances needed to enable effective spoken language interaction with robotics; little attention was given to communicative failures. To our knowledge, the documentation of failure in speech interfaces and systematic studies of such failures and their causes is exceedingly rare. Honig and Oron-Gilad (2018) provides the most in-depth literature review of prior failure-related HRI studies. The authors found that research in HRI has focused mostly on technical failures, with few studies focusing on human errors, many of which are likely to fall under the umbrella of conversational failures. In addition to this focus on technical errors, the majority of failure-related studies in HRI take place in controlled experimental conditions, where “failures” are explicitly designed and occur only at specific moments (Ragni et al., 2016; Washburn et al., 2020a; Cuadra et al., 2021; Green et al., 2022), instead of a natural occurrence of the interactions between humans and robots. Closer to the topic of the workshop is the recently proposed taxonomy of Tian and Oviatt (2021) that focuses on social errors in HRI and their relationship with the perceived socio-affective competence of a robot. However, while there is significant overlap between social errors, as categorized by Tian and Oviatt, and the workshop topic of conversational failure, the perspective on the role of these errors and failures in interaction as well as the view as to whether these could be overcome eventually differs significantly. While social errors should ultimately be reduced by increasing a robot’s perceived socio-affective competence, it appears unlikely that conversational failure could be totally extinguished by means of technological progress. Too frequent is their occurrence in human-human conversation and too deeply ingrained are the related repair mechanisms in the fabric of human communication.

To the best of our knowledge, there are currently no survey papers specifically on conversational failures in human-robot interaction, a fact that illustrates an important gap in the research landscape. To address this gap, we conducted a two-phase workshop with experts in adjacent fields. This paper presents the findings from this workshop series that brought together a multidisciplinary group of researchers from fields such as robotics, human-robot interaction (HRI), natural language processing (NLP), conversation analysis, linguistics and pragmatics. The workshop provided a platform to discuss the multitude of failures of speech interfaces openly and to point out fruitful directions for overcoming these failures systematically. The workshop focused mainly on human-robot joint action scenarios involving multimodal coordination between humans and robots, as these are the norm in scenarios where robotic speech interfaces are deployed. The identified types of failures range from failures of speech recognition to pragmatic failures and infelicities.

We begin by describing the aims, structure, and materials used in the workshop in Section 2. We then present findings that result from the workshop, including participant contributions and outcomes of the structured discussion in Sect. 3. This leads to Sect. 4, where we reflect on problems and identify themes that emerged from the workshop’s discussions before concluding the paper.

## 2 Materials and methods

The *Working with Troubles and Failures (WTF) in Conversations between Humans and Robots* workshop included a virtual gathering over two consecutive days in June 2022 and an in-person full-day meeting at the University of Hertfordshire in September 2022. Here, we sketch the structure and summarize the findings for each of these parts.

### 2.1 Before the workshop

In order to attract workshop participants interested in an open discussion of their experience and investigations of failing speech interfaces, we directly contacted some of the potentially interested research groups within the United Kingdom. Additionally, the workshop was advertised via mailing lists relevant to the HRI (e.g., *hri-announcement*, *robotics-worldwide*, *euRobotics-dist*), natural language processing (NLP, e.g., *ACM sigsem*), and artificial intelligence communities (e.g., ACM *sigai-announce*). To verify participants’ genuine interest in the topic and to collate information on the different types of conversational failures experienced by them, they were asked to submit the following pieces of information.

1. The number of years of experience using or developing speech interfaces,
2. An indication of what they perceive to be the most pressing issue or the biggest source of failure for speech interfaces,
3. Their most memorable WTF moment, that is, which of their experiences of failure with a speech interface they remembered most vividly,
4. A summary of their motivation to attend the workshop,
5. A suggestion for a future benchmark scenario that would expose the kind of failure described in their WTF moment.

Applicants that stated a meaningful entry for item 4, and made some attempt to answer the other questions, were admitted to the workshop. As a result, 15 participants were admitted and initially attended the virtual part. Of these fifteen participants, eight would go on to attend the face-to-face part of the workshop. The face-to-face workshop was re-advertised via the above-mentioned mailing lists and the same set of questions and answers was used to filter out additional prospective participants. Ultimately, six new participants joined the face-to-face part of the workshop, resulting in fourteen non-speaker, non-organiser participants. Two of these attended the face-to-face workshop virtually, as we decided to go for a hybrid format in order not to exclude anyone who was not able or willing to travel on site.

Keynote speakers for both parts of the workshop were chosen based on their expertise in the subject area. The subject areas considered most relevant to the workshop were robotics-centred NLP on the one hand and Conversation Analysis (CA) on the other. The emphasis on CA was based on the fact that the documentation and analysis of conversational failure have been an integral part of this discipline since its very inception. Moreover, it was hoped that having keynote speakers and participants from both areas would soften discipline-specific boundaries and limitations and potentially open up new directions for future research.

#### 2.1.1 Motivations for attending the workshop

The following is a summary of the participants’ motivation for attending the workshop as extracted from the application forms.

Several PhD students were hoping to connect and network with other researchers working in speech interaction technologies. Multiple other researchers working on the CA-HRI interface wanted to learn more about how conversational trouble emerges, while others occupied with developing speech interfaces, or with integrating these into robots were interested in gaining a deeper understanding of current issues. Many of them were also interested in sharing their experiences with peers.

One researcher working in animal communication hoped to learn something from a different domain of “inter-being communication”, while yet another researcher working on speech privacy wanted to connect to other researchers working on speech interfaces. One participant saw value in the aim of identifying or creating a benchmark scenario that would be able to tease out the most common failures, if they occurred - an aim explicitly set out by the workshop.

Another motivation of multiple participants to attend the workshop was their shared belief that a deeper analysis of communicative failures would not only help to improve future speech interfaces but also gain a deeper understanding of (human) conversations themselves.

Finally, a researcher interested in explainable AI was interested to see what other types of failures, apart from faulty explanations, there are and how these may connect to research in explainable AI.

### 2.2 Virtual workshop

To facilitate participation in the virtual session of the workshop, it was divided into two half-day events. On the first day, the workshop opened with a keynote talk by Prof. Patrick Healey, Professor of Human Interaction and Head of the Cognitive Science Research Group in the School of Electronic Engineering and Computer Science at Queen Mary University of London, on “Running repairs: Coordinating meaning in dialogue” (Section 3.1.1). This was followed by participants’ lightning talks on their most memorable WTF moments when working with communication between humans and robots (Section 3.2). Following the lightning talks, and based on the underlying themes identified by the organisers, participants were divided between 4 breakout rooms to continue discussing the issues they brought to the workshop. The four identified themes were: i) Context Understanding, ii) Handling Miscommunication, iii) Interaction Problems, and iv) General Failures.

The second day of the virtual workshop saw Dr. Saul Albert, Lecturer in Social Science (Social Psychology) in Communication and Media at Loughborough University, give a keynote talk on “Repair, recruitment, and (virtual) agency in a smart homecare setting” (Section 3.1.2). Following the talk, each group from the breakout rooms of the first day reported what was discussed and each debate was opened to all participants. The workshop ended with a short summary of the day.

### 2.3 Face-to-face workshop

The in-person part of the workshop was held at the University of Hertfordshire 3 months after the virtual event. During this full-day meeting, keynote talks were given by Prof. Gabriel Skantze, Professor in Speech Technology at KTH Royal Institute of Technology on “Building Common Ground in Human-Robot Interaction” (Section 3.1.3) and by Dr. Ioannis Papaioannou, Chief Technology Officer & Co-Founder of Alana^1^ on “Tackling the Challenges of Open-Domain Conversational AI Systems” (Section 3.1.4).

Since the registration to the face-to-face workshop was also opened to participants who did not take part in the virtual workshop, new attendees were given the opportunity to present their own lightning talks on their WTF moments (Section 3.2).

A central part of the face-to-face workshop was the World Café session^2^, which provided participants an opportunity to freely discuss troubles and failures in small groups across several table topics. Based on the participants’ submitted WTF moments, and the themes from the breakout rooms of the virtual part, four themes were chosen for this session: i) Context Understanding, ii) Interaction Problems, iii) Handling Miscommunication, and iv) Suggested Benchmark Scenarios. Each theme was allocated to one table, and each table had one designated organizer. Participants and speakers were split into four different groups and moved between the tables within time slots of approximately 15 min per theme. The tasks of a table’s organizer were to summarize the findings and discussions from previous groups to a newly arriving group, to encourage discussions around the table topic, and to either encourage note taking or take notes themselves on a large flip chart that was allocated to each table.

## 3 Results

In this section, we present findings from both the virtual and the face-to-face parts of the workshop, describing how the keynotes shaped the discussion and how the participant lightning talks contributed to identify some of the most pressing problems in conversations between humans and robots. Most importantly, we will present the outcomes of the structured discussion, summarising the workshop findings.

### 3.1 Keynotes

To frame the discussion on troubles and failures with experiences from different perspectives, we invited four keynote speakers from scientific areas that are concerned with research problems around conversations between humans and robots. This section summarises their presentations in the context of the workshop goals to scope and identify common troubles and failures in conversation between humans and robots. In the virtual part of the workshop, the first keynote (Sect. 3.1.1) provided a conversation analytical perspective on repairs and meaning in dialogue, while the second one looked at repairs but from a more applied perspective in a user’s home (Sect. 3.1.2). The in-person workshop provided insights considering human-robot interactions (Sect. 3.1.3) and an industry viewpoint (Sect. 3.1.4).

#### 3.1.1 Running repairs: coordinating meaning in dialogue

Healey presented the Running Repairs Hypothesis (Healey et al., 2018b), which captures the idea that successful communication depends on being able to detect and adjust to misunderstandings on the fly. The basic assumption is that no two people ever understand exactly the same thing by the same word or gesture and, as a result, misunderstandings are ubiquitous. Data from conversations support this assumption. For example, the utterance “huh?” occurs around once every 84 s in conversation and appears to be universal across human languages (Dingemanse et al., 2015; Enfield, 2017). Around a third of turns in ordinary conversation involve some sort of real-time adjustments in language use (Colman and Healey, 2011).

The processes for detecting and resolving problems with understanding have conventionally been regarded as “noise in the signal” by the cognitive sciences (Healey et al., 2018a). However, there is evidence that they are fundamental to our ability to adapt, in real-time, to new people, new situations and new tasks. Conversation analysts have described a set of systematic turn-based *repair* processes that structure how people identify and respond to misunderstandings (Schegloff et al., 1977a; Schegloff, 1992a; Schegloff, 1997). Experimental evidence shows these repair processes have a critical role in building up shared understanding and shared languages on the fly (Healey, 2008; Healey, 1997; Healey et al., 2018b).

The Running Repairs Hypothesis characterises human communication as a fundamentally error-prone, effortful, active, collaborative process but also highlights how these processes are structured and how they make human communication flexible and adaptable to new people and new situations. This can liberate human-robot interaction from the fantasy of perfect competence (Park et al., 2021). Instead, robots could, in principle, take advantage of the resources of interaction by engaging in repairs. This requires developing the ability to recognise critical verbal and non-verbal signals of misunderstanding and the use of incremental online learning processes that build on the sequential structure of interaction to make real-time revisions to language models (see e.g., Purver et al., 2011; Howes and Eshghi, 2021).

#### 3.1.2 Repair, recruitment, and (virtual) agency in a smart homecare setting

Albert argued that moments of trouble and failure can provide researchers with ideal empirical material for observing the structure of the participation frameworks we use to get things done in everyday life (Goodwin, 2007; Albert and Ruiter, 2018). His presentation used multimodal video analysis to show how a disabled man and his (human) carer leveraged troubles and failures in their interactions with an Amazon Echo with voice-controlled lights, plugs, and other devices to co-design an effective smart homecare participation framework.

Instances in this case study highlighted how the human carer used troubles and failures to prioritise the independent role and agency of the disabled person within a joint activity. For example, the carer would stop and wait for the disabled person to resolve the trouble in their interactions with the virtual agent and complete their task even when it would have been faster for the carer to complete the disabled person’s task manually. In other examples, trouble in the interactions between the carer and the virtual assistant provided an opportunity for the disabled person to intervene and assist the carer by correcting and completing their vocal instruction to the device. The disabled person was also able to tacitly “recruit” (Kendrick and Drew, 2016) assistance from the human carer by repeatedly re-doing failed commands to the virtual assistant within earshot of the carer, soliciting support without having to ask for help directly.

These episodes show how people can harness trouble and failures in interaction with a virtual assistant to enable subtle shifts of agency and task-ownership between human participants. This kind of hybrid smart homecare setting can support and extend the independence of a disabled person within an interdependent, collaborative participation framework (Bennett et al., 2018). More broadly, the communicative utility of trouble and failure in interactions with machines highlights the shortcomings of our idealized–often ableist–models of the “standard” user, and medicalized models of assistive technology (Goodwin, 2004; Albert and Hamann, 2021).

#### 3.1.3 Building common ground in human-robot interaction

Skantze highlighted two aspects of miscommunication and error handling in human-machine interaction. First, he discussed how language is ultimately used as part of a joint activity. For communication to be meaningful and successful, the interlocutors need to have a mutual understanding of this activity, and of their common ground (Clark, 1996). From this perspective, language processing is not a bottom-up process, where we first figure out what is being said before interpreting and putting it in context. Rather, we use the joint activity to steer the interpretation process and possibly ignore irrelevant signals. Skantze exemplified this with an early experiment, where a noisy channel (including a speech recognizer) was used in a human-human communication task, where one person had to guide another person on a virtual campus (Skantze, 2005). Although much of what was said did not get through (due to the error prone speech recognition), the humans very seldom said things like “sorry, I did not understand”, which are frequent responses in human-machine interactions. Instead, they relied on the joint activity to ask task-related questions that contributed to task progression. Another implication of this view on communication is that the idea of “open-domain dialogue”, where there is no clear joint activity, is not meaningful to pursue (Skantze and Doğruöz, 2023).

The second aspect that was discussed was the need to incorporate user feedback when the system is speaking, and use that feedback to model what can be regarded as common ground between the user and the system. Skantze exemplified this issue with a research project at KTH (Axelsson and Skantze, 2023), where an adaptive robot presenter is being developed (in the current demonstrator it is talking about classic works of art in front of a human listener). The robot presenter uses a knowledge graph to model the knowledge it is about to present, and then uses that same graph to keep track of the “grounding status” of the different pieces of information (Axelsson and Skantze, 2020). Multimodal feedback from the user (e.g., gaze, facial expressions, nods and backchannels) are interpreted as negative or positive, and the graph is updated accordingly, so that the presentation can be adapted to the user’s level of knowledge and understanding (Axelsson and Skantze, 2022).

#### 3.1.4 Addressing the challenges of open-domain conversational AI systems

Papaioannou’s presentation showed how designing conversational AI systems able to engage in open-domain conversation is extremely challenging and a Frontier of current research. Such systems are required to have extensive awareness of the dialogue context and world knowledge, the user intents and interests, requiring more complicated language understanding, dialogue management, and state and topic tracking mechanisms compared to traditional task-oriented dialogue systems.

In particular, some of these challenges include: (a) keeping the user engaged and interested over long conversations; (b) interpretation and generation of complex context-dependency phenomena such as ellipsis and anaphora; (c) mid-utterance disfluencies, false starts, and self-corrections which are ever-present in spoken conversation (Schegloff et al., 1977b; Shriberg, 1994) (d) various miscommunication and repair phenomena such as Clarification Requests (Purver, 2004) and Third Position Repair (Schegloff, 1992b) whereby either the user or system does not understand the other sufficiently or misunderstands, and later repairs the misunderstanding. (b-d) Are all crucial to robust Natural Language Understanding in dialogue.

A modular conversational AI system (called *Alana*), tackling some of the aforementioned challenges (i.e., user engagement over long conversations, ellipsis and anaphora resolution, and clarification requests) was developed between 2017 and 2019 (Papaioannou et al., 2017; Curry et al., 2018) and deployed to thousands of users in the United States as part of the Amazon Alexa Challenge (Ram et al., 2018). The Alana system was also evaluated in a multimodal environment and was used as the overall user conversational interaction module in a multi-task and social entertainment robotic system as part of the MuMMER project (Foster et al., 2019). The integrated system was deployed in a shopping mall in Finland and was able to help the user with specific tasks around the mall (e.g., finding a particular shop or where they could buy a certain product, finding the nearest accessible toilet, or asking general questions about the mall) while at the same time engaging in social dialogue and being entertaining.

The output of that research was fed to the implementation of the “Conversational NLU” pipeline by Alana AI, a modular neuro-symbolic approach further enhancing the language understanding of the system. The Conversational NLU module is able to detect and tag a number of linguistic phenomena (e.g., disfluencies, end-of-turn, anaphora, ellipsis, pronoun resolution, etc.) as well as detect and repair misunderstandings or lack of sufficient understanding, such as self-repairs, third-position corrections, and clarifications. The system is currently being evaluated by blind and partially sighted testers in the context of multi-modal dialogue allowing the users to find mislocated objects in their environment via a mobile application.

### 3.2 Lightning talks

The following section contains short summaries of the lightning talks of both the virtual and the face-to-face part of the workshop. From the presentations, three themes were identified: *Description and Analysis of Failures and Troubles* (Sect. 3.2.1) grouping presentations that have a descriptive or analytical focus; *Technical Aspects of Conversational Failure* (Sect. 3.2.2) for presentations that have a more technical focus; and *Adjacent Topics in Speech Interfaces* (Sect. 3.2.3), grouping presentations on topics that, while not focusing strictly on conversational failures, covering other forms of errors and issues that fall into the wider topic of speech-centric human-machine interactions. Note that many of the talks falling into the second, technical category still contain a substantial element of analysis that enabled or inspired the technical solutions described therein.

#### 3.2.1 Description and analysis of failures and troubles

The following ten of the contributions took a more analytical approach to the failure they reported in their lightning talks. They describe possible reasons or implications of the failure they present.

##### 3.2.1.1 Laundrobot: Learning from human-human collaboration

Barnard and Berumen presented their work on *Laundrobot*, a human acting as a collaborative robot designed to assist people in sorting clothing into baskets. The study focused on participants’ ability to collaborate through verbal instructions and body movements with a robot that was sometimes erroneous when completing the task. The team analysed social signals, including speech and gestures, and presented three cases demonstrating human-human collaboration when things do not go as expected. In one of the cases, a participant gave clear instructions to an erroneous Laundrobot, which led to frustration on the participant’s part, with statements such as “Okay, I’m doing this wrong”. The presenters described how the participant appeared to take responsibility for the errors made by the robot. They examined the use of language and expression of intent in different instances for pieces of clothing that were either correctly or incorrectly identified by Laundrobot. During this analysis, Barnard, Berumen, and others came across an interesting case regarding the use of the word “right”, which was frequently used in both erroneous and non-erroneous instances. The group explored how that word had different meanings depending on the success or failure of Laundrobot. For instance, for one participant (P119), the word had a single meaning of indicating a direction in erroneous instances, whereas, on other occasions, it had alternative purposes. It was sometimes used to refer to directions and, at other times, used for confirmation, immediacy (“right in front of you”), or purpose (“Right, OK”).

##### 3.2.1.2 Sequential structure as a matter of design and analysis of trouble

As part of the *Peppermint project* ^3^ corpus, Tisserand presented a transcript fragment, reproduced below. They designed a Pepper robot as an autonomous reception desk agent that would answer basic requests asked by library users. They captured *naturally-occurring interactions*: the robot was placed in the library, and users were free to interact and leave whenever they wanted.

01 Hum: where can I find books of maths?

| Sequence A - Part 1

02 Rob: ((provides the direction for books of maths))

| Sequence A - Part 2

03 Rob: is it clear to you?

| Sequence B - Part 1

04 Hum: yes thanks

| Seq B-2 && Seq A-3

05 Rob: okay, I will repeat ((repeats turn line 2))

| Sequence C - Part 1

The failure here is the fact that the robot recognized “no thanks” instead of two separate actions: “yes” + “thanks” (l.4); the robot thus repeats the answer to the user’s question. Reflecting on this WTF moment, Tisserand highlighted how this failure occurred due to decisions made during the scenario design phase. Firstly, poor speech recognition differentiation between the words “yes” and “no” had led the scenario design team to add “no thanks” to a word list provided for recognising an *offer rejection* (a *dispreferred turn design* for this type of action (Schegloff, 2007, Chap.5)) in another scenario in which the robot makes an offer. Secondly, because the state machine was based on isolated so-called “contexts”, it was designed only to make one decision when processing a spate of talk. Here, therefore, the clarification check turn in line 3 was treated as independent from the question response in line 2. Because the speech recognition system struggled to differentiate “yes” and “no”, and was using the word list that labelled “no thanks” as a case of*offer rejection*, here it erroneously recognized “yes thanks” in line 4 as a negation (a *clarification denial*), and proceeded to repeat the turn.

What should have happened is that when the robot asks the user to confirm (l.3), it should recognize that this sequence is embedded in the previous question/answer sequence (l.1–2). In this case, the human’s “yes” (l.3) is a response to the just-prior confirmation request while the “thanks” responds (in the first structurally provided sequential slot) to the Robot’s answer as a “sequence closing third” (l.3). This is why the team is now *sequentially* annotating training datasets to show what utterances correspond not only to questions and answers, but also the cement in-between: how the user might delay, suspend, abandon, renew or insert actions (e.g., repair). Here interaction is seen as a temporally continuous and incremental process and not a purely logical and serial one. In other words, context is seen as an organized resource more than an adaptability constraint.

##### 3.2.1.3 Design a Robot’s spoken behaviours based on how interaction works

Huang pointed out that spoken interaction is complicated. It is grounded in the social need to cooperate (Tomasello, 2009; Holtgraves, 2013) and requires interlocutors to coordinate and build up common ground on a moment-by-moment basis (Krauss and Fussell, 1990, p.112) (Holtgraves, 2013).

Speech is only one tool in a larger picture. Some errors are caused by failures in natural language understanding (NLU) as illustrated in the following sequence:

01 User: Let’s talk about me.

02 Robot: What do you want to know about ‘me’?

Other issues, however, could be caused by a lack of understanding of common ground. For example, when a naive user asked, “Where to find my Mr Right”, the system provided a place named “Mr & Mrs Right” and told the user it was far away. This reply contains several layers of failure: 1) the robot fails to capture the potential semantic inference of the expression *Mr Right*; 2) it fails to consider the social norm that Mr Right belongs typically to one person only; and 3) it makes a subjective judgement about distance. One may argue that this error would not happen if the user knew a question-answer robot could not chat casually. However, the issue is whether a clear boundary of a social robot’s capability is set in the system or communicated to the user during the interaction. It is difficult to tell why speech interfaces may fail and how to work around the limits without understanding what makes interaction work and how speech assists in the process.

Also, spoken interaction requires interlocutors, including robots, to adjust their behaviours based on the verbal and non-verbal feedback provided by others. A social robot that does not react appropriately could be deemed improperly functional, as illustrated in the following sequence. In the scenario, the robot failed to generate satisfactory answers several times in an open conversation; the user felt frustrated.

User: You are generating GPT rubbish.

Robot: (No response, carries on)

##### 3.2.1.4 Hey Siri … you Don’t know how to interact, huh?

The WTF moment Wiltschko presented concerned the use of *huh* in interaction with Siri, Apple’s voice assistant.

User: Hey Siri, send an e-mail.

Siri: To whom shall I send it?

User: huh?

Siri: I couldn’t find huh in your contacts. To whom shall I send it?

It is evident from the example that Siri cannot understand *huh*. This is true for *huh* used as an other-initiated repair strategy as in the example above, but it is also true for its use as a sentence-final tag. This is a significant failure as in human-human interaction the use of *huh* is ubiquitous. In fact, *huh* as a repair strategy has been shown to be available across a number of unrelated languages (Dingemanse et al., 2013). Wiltschko speculates that successful language use in machines is restricted to propositional language (i.e., language used to convey content) whereas severe problems arise in the domain of interactional language (i.e., language used to regulate common ground building as well as the conversational interaction itself). The question that arises, however, is whether human users feel the need to use interactional language with machines. After all, this aspect of language presupposes interaction with another mind for the purpose of common ground construction and it is not immediately clear whether humans treat machines as having a mind with which to share a common ground.

##### 3.2.1.5 Utilising explanations to mitigate robot failures

Kontogiorgos presented current work on failure detection (Kontogiorgos et al., 2020a; Kontogiorgos et al., 2021) and how robot failures can be used as an opportunity to examine robot explainable behaviours. Typical human-robot interactions suffer from real-world and large-scale experimentation and tend to ignore the “imperfectness” of the everyday user (Kontogiorgos et al., 2020b). Robot explanations can be used to approach and mitigate robot failures by expressing robot legibility and incapability (Kwon et al., 2018), and within the perspective of common-ground. The presenter discussed how failures display opportunities for robots to convey explainable behaviours in interactive conversational robots according to the view that miscommunication is a common phenomenon in human-human conversation and that failures should be viewed as being an inherent part of human-robot communication. Explanations, in this view, are not only justifications for robot actions, but also embodied demonstrations of mitigating failures by acting through multi-modal behaviours.

##### 3.2.1.6 Challenging environments for debugging voice interactions

Porcheron presented the challenge of how we expect users to understand and debug issues with “eyes-free voice interactions”, and of parallelism to the prospects of voice-based robots. A recurrent promise of voice-based technologies is their simplicity: we issue a command to a computer and it can respond accordingly. Of course, not all technology use goes as planned and sometimes errors occur. With graphical user interfaces (GUIs), we have a plethora of well-tested heuristics (e.g., Nielsen, 1995), especially for dealing with “errors” where users need “fix” something. However, with voice, in situations where people encounter something going wrong, they have to carry out work to figure out how to resolve the issue (Porcheron et al., 2018; Fischer et al., 2019). One specific example is responses which do not reveal specifics, such as “I had an issue responding to that request”. Users are given little purchase with which to debug this issue, and attempt to resolve this. This user challenge is exacerbated in the new settings where voice technologies are appearing: in our cars, on our bikes, and anywhere we take our smartwatch—in these settings, there is often little time to read and respond to a text, little audible information to go on, and plenty of distraction for the user. Porcheron suggested that if we want to consider voice as a modality for controlling robots, we first need to think through how we help users understand and recover from “errors” in these sorts of environments first.

##### 3.2.1.7 The robot [does not] hear me correctly

Hernández García presented examples of failures of user interactions with a receptionist robot. Gunson et al. (2022) developed a social conversational robot that can act as a receptionist in a hospital waiting room for tasks such as greeting visitors, answering common questions, providing directions and/or scheduling information, *etc.* During the evaluation with users (student participants in a lab setting), a majority of failed interactions that prevented user task completion were the result of problems with the ASR. These ASR failures predominately took the form of an ASR result that contained only part of the user’s utterance, misrecognition of critical words, or a combination of the two (to the extent that the user’s original intention could not be discerned), leading the dialogue management into a non-coherent path and frustrating the user.

USER: can you help me to find the exit please | ASR: please

ROBOT: Anyway it’s a big building we’re in, isn’t it. Did you know you can ask me for help if you ever get lost here?

USER: can you direct me to the exit please | ASR: can you direct me to Netflix please

ROBOT: Such a lot of waiting around isn’t there.

USER: thank you [user left]

Participants rated the conversation negatively when these errors occurred, as they experienced difficulties in making themselves understood. The user evaluations reported by Gunson et al. (2022) highlighted that users did not feel it was *natural* or that it *flowed* in the way they expected. Participants did not believe that “*the robot heard me correctly most of the time*” or that “*the robot recognised the words I said most of the time*” nor “*felt confident the robot understood the meaning of my words*”.

Conversational troubles may start at a *speech recognition* level, but these failures are propagated throughout the whole *speech interface* pipeline, compounding to create WTF moments and leading to poor performance, increasing user frustration, and loss of trust, *etc.*

##### 3.2.1.8 Hello, It is nice to “meat” you

Nesset shared examples of WTF moments encountered while interacting with Norwegian chatbots through written text. The first failure presented was users’ committing spelling mistakes interacting with a virtual agent through chat. This caused the agent to misunderstand the overall context of the conversation. A good example of this is misspelling meet with meat, and the chatbot then replying with a response about sausages.

The second part entailed a user failure that is specifically for multilingual users. In some non-native English-speaking countries, such as Norway, technical terms and newer words are often commonly said in English. This potentially leads users to interact with agents in two languages within the same sentence/conversation. This can lead to the agent struggling to interpret the terms in the second language, and assuming that they mean something else in the original interaction language. These are some examples of how uncertain user output can result in failures from the robot.

##### 3.2.1.9 Speech misrecognition: a potential problem for collaborative interaction in table-grape vineyards

Kaszuba presented troubles and failures encountered while designing a spoken human-robot interaction system for the *CANOPIES project* ^4^. This project aims to develop a collaborative paradigm for human workers and multi-robot teams in precision agriculture, specifically in table-grape vineyards. When comparing some already existing speech recognition modules (both online and offline), the presenter identified communication issues associated with the understanding and interpretation of specific words of the vineyard scenario, such as “grape”, “bunch”, and “branch”. Most of the tested applications could not clearly interpret such terms, leading the user to repeat the same sentence/word multiple times.

Hence, the most significant source of failure in speech interfaces that Kaszuba has described is *speech misrecognition*. Such an issue is particularly relevant, since the quality and effectiveness of the interaction strictly depend on the percentage of words correctly understood and interpreted. For this reason, the choice of the application scenario has a crucial role in the spoken interaction, and preliminary analysis should be taken into consideration when developing such systems, as the type and position of the acquisition device, the ambient noise and the ASR module to adopt. Nevertheless, misrecognition and uncertainty are unavoidable when the developed application requires people to interact in outdoor environments and communicate in a language that is not the users’ native language.

Hence, some relevant considerations concerning ASR modules should be taken into account in order to implement a robust system that, eventually, can also be exploited in different application scenarios. The percentage of uncertainty, the number of misrecognized words and the environmental noise that can negatively affect communication are some fundamental issues that must be addressed and minimized.

##### 3.2.1.10 Leveraging multimodal signals in human motion data during miscommunication instances

Approaching from a natural dialogue standpoint and inspired by the Running Repairs Hypothesis (Healey et al., 2018b), Özkan shared a presentation on why and how we should take advantage of WTF-moments or miscommunications to regulate shared understanding between humans and speech interfaces. Rather than avoiding these moments (which is impossible), if speech interfaces were to identify them and show appropriate behaviour, it could result in more natural, dynamic and effective communication.

Detecting miscommunications from the audio signal can only can be costly in terms of computational load or prone to error due to noise in most environments. Fortunately, repair phenomena manifest themselves in non-verbal signals as well (Healey et al., 2015; Howes et al., 2016). Findings regarding speaker motion during speech disfluencies (self-initiated self-repairs) have shown that there are significant patterns in the vicinity of these moments (Özkan et al. 2021; Özkan et al., 2023; Ozkan et al., 2022). Specifically, the speakers have higher hand and head positions and velocities near disfluencies. This could be treated as a clear indicator for artificial interfaces to identify troubles of speaking in their human partner. For example, to the user input “*Could you check the flights to Paris -uh, I mean- Berlin?*”, the interface, instead of disregarding the uncertain utterance, could offer repair options more actively by returning “*Do you mean Paris or Berlin?*” in a collaborative manner.

Though not in the context of disfluencies, a common example of not allowing repair (in this case other initiated other repair) occurs when the user needs to correct the output of an interface or simply demand another response to a given input. As a WTF moment in the repair context, Özkan demonstrated a frequent problem in their interaction with Amazon Alexa. When asked to play a certain song, Alexa would play another song with the same or similar name. The error is not due to speech recognition, because Alexa understands the name of the song very well. However, it maps the name to a different song that the user does not want to hear. No matter how many times the user tries the same song name input, even with the artist name, Alexa would still pick the one that is the “first” result of its search. If the conversational repair was embedded in the design, a simple solution to this problem could have been “*Alexa, not that one, can you try another song with the same name?*”, but Alexa does not respond to such requests.

#### 3.2.2 Technical Aspects of Conversational Failure

The following five of the contributions describe technical aspects of failures. Presentations in this section either discuss the technical causes of failures, point out technological attempts to recognize when conversational trouble occurs, or summarize approaches on handling troubles on part of the robot.

##### 3.2.2.1 Chefbot: Reframing failure as a dialogue goal change

Gkatzia presented their work on *Chefbot*, a cross-platform dialogue system that aims to help users prepare recipes (Strathearn and Gkatzia, 2021a). The task moves away from classic instruction giving and incorporates question-answering for clarification requests, and commonsense abilities, such as swapping ingredients and requesting information on how to use or locate specific utensils (Strathearn and Gkatzia, 2021b). This results in altering the goal of the communication from cooking a recipe to requesting information on how to use a tool, and then returning to the main goal. It was quickly observed that changing the dialogue goal from completing the recipe to providing information about relevant tasks resulted in failure of task completion. This issue was subsequently addressed by *reframing* failure as a temporary dialogue goal change, which allowed the users to engage in question answering that was not grounded to the recipe document, and then forcing the system to resume the original goal.

##### 3.2.2.2 Failure in speech interfacing with local dialect in a noisy environment

Liza (Farhana) presented their ongoing work in capturing the linguistic variation of speech interfaces in real-world scenarios. Specifically, local dialects may impose challenges when modelling a speech interface using an artificial intelligence (deep learning) language modelling system. Deep learning speech interfaces rely on language modelling which is trained on large datasets. A large dataset can capture some linguistic variations; however, dialect-level variation is difficult to capture as a large enough dataset is unavailable. Moreover, very large models require high-performance computation resources (e.g., GPU) and take a long time to respond, which imposes further constraints in terms of deploying such systems in real scenarios. Large data-driven solutions also cannot easily deal with noise as it is impractical to give access to enough real-world data from noisy environments. Overall, state-of-the-art AI models are still not deployable in scenarios with dialect variation and noisy environments. Alharbi et al. (2021) identified several hurdles in training end-to-end Automatic Speech Recognition (ASR) models. Additionally, the conditional interdependence between the acoustic encoder and the language model was emphasized by Xu et al. (2020). Consequently, while augmenting the standard text training data can enhance the efficacy of general-purpose language models, the limited availability of corresponding acoustic data poses challenges in training end-to-end ASR systems. Moreover, when addressing dialect modeling (Hirayama et al., 2015), the scarcity of training data exacerbates the difficulties in integrating speech interfacing and language modeling (Liza, 2019) within the ASR framework.

##### 3.2.2.3 The “W” in WTF moments can also be “when”: The importance of timing and fluidity

Hough presented WTF moments driven more by inappropriate timing of responses to user utterances, rather than by content misunderstandings. Improving the first-time accuracy of Spoken Language Understanding (SLU) remains a priority for HRI, particularly given errors in speech recognition, computer vision and natural language understanding remain pervasive in real-world systems. However, building systems capable of tolerating errors whilst maintaining *interactive fluidity* is an equally important challenge. In human-human situated interactions where an instructee responds to a spoken instruction like “put the remote control on the table” and a follow-up repair like “no, the left-hand table” when the speaker realizes the instructee has made a mistake, there is no delay in reacting to the initial instruction, and adaptation to the correction is instant (Heldner and Edlund, 2010; Hough et al., 2015), in stark contrast to state-of-the-art robots with speech interfaces. Increasing interactive fluidity is vital to give robots with speech understanding more seamless, human-like transitions from processing speech to taking physical action without delay, permitting appropriate overlap between the two, and the ability to repair actions in real-time. Rather than waiting for components to be perfected, preliminary experiments with a pick-and-place robot show users can be tolerant of errors if fluidity is kept high, including appropriate repair mechanisms (Hough and Schlangen, 2016).

##### 3.2.2.4 Laughter in WTF moments

Maraev presented a hypothesis that laughter can be treated as an indicator of a WTF moment. Laughter can occur in such moments as a) speech recognition failures disclosed to a user via explicit grounding feedback, b) awkwardness due to retrieval difficulties, c) resulting system apologies and down players (e.g., “do not worry”). Along with examples from task-oriented role-played dialogues, Maraev discussed the following constructed example, where laughter communicates a negative feedback to the system’s clarification of speech recognition result:

Usr> I would like to order a vegan bean burger.

Sys> I understood you’d like to order a vegan beef burger. Is that correct?

Usr> HAHAHA

Maraev et al. (2021) focused on non-humorous laughs in task-oriented spoken dialogue systems. The paper shows how certain types of laughter can be processed within the dialogue manager and natural language generator, namely,: laughter as negative feedback, laughter as a negative answer to a polar question and laughter as a signal accompanying system feedback.

##### 3.2.2.5 To err is robot

Giuliani presented findings from 6 years of research on erroneous human-robot interactions. The team of researchers led by Giuliani has shown that participants in human-robot interaction studies show unique patterns of social signals when they experience an erroneous situation with a robot (Mirnig et al., 2015). The team annotated two large video corpora of 201 videos showing 578 erroneous situations and 1,200 videos showing 600 erroneous situations, respectively (Giuliani et al., 2015; Cahya et al., 2019). They found that there are two types of errors that do occur in human-robot interaction. Social norm violations are situations in which the robot does not adhere to the underlying social script of the interaction. Technical failures are caused by the technical shortcomings of the robot. The results of the video analysis show that the study participants use many head movements and very few gestures but they often smile when in an error situation with the robot. Another result is that the participants sometimes stop moving at the beginning of error situations. The team was also able to show in a user study for which a robot was purposefully programmed with faulty behaviour that participants liked the faulty robot significantly better than the robot that interacted flawlessly (Mirnig et al., 2017). Finally, the team trained a statistical model for the automatic detection of erroneous situations using machine learning (Trung et al., 2017). The results of this work demonstrate that automatic detection of an error situation works well when the robot has seen the human before.

#### 3.2.3 Adjacent Topics in Speech Interfaces

The two contributions under this theme do not discuss conversational failures directly but address the related topics of explanatory AI and privacy of speech interfaces.

##### 3.2.3.1 What is a “good” explanation?

Kapetanios presented some thoughts around the long-standing research question of *what is a good explanation* in the context of the current buzz around the topics of explainable AI (XAI) and interpretable Machine Learning (IML). Using Amazon’s Alexa and Google’s Digital Assistant to generate explanations for answers being given to questions being asked of these systems, he demonstrated that both systems, at the technological forefront of voice-based HCI approaches to answering specific questions, fail to generate convincing explanations. Convincing explanations should fit the facts, be relevant, tailored to the recipient, and typically do more than merely describe a situation (Dowden, 2019, chap. 14). It is frequently the latter where digital assistants have been observed to struggle. Hence, when describing the results of running several thousand queries through the most common digital assistants, provides the following example (Enge, 2019).

Siri, when being asked the question “Who is the voice of Darth Vader?”, instead of providing the name of the (voice) actor, returns a list of movies featuring Darth Vader. While this answer is topically relevant, it certainly is not a proper answer to the question. The same problem of explanation persists with ChatGTP-3/4, despite its fluency in generating precise answers to specific questions in natural language.

##### 3.2.3.2 Privacy and security issues with voice interfaces

Williams presented privacy and security issues and how these are often underestimated, overlooked, or unknown to users who interact with voice interfaces. What many voice interface users are unaware of is that only three to 5 seconds of speech are required to create a *voiceprint* of a person’s real voice as they are speaking (Luong and Yamagishi, 2020). One of the risks that follows is that voiceprints can be re-used in other voice applications to impersonate or create voice deepfakes (Williams et al., 2021b; Williams et al., 2021a). In the United Kingdom and many other countries, this poses a particular security risk as voice-authentication is commonly used for telephone banking and call centres. In addition, some people may be alarmed when a voice interface reveals private information by “speaking out loud” sensitive addresses, birth dates, account numbers, or medical conditions. Anyone in the nearby vicinity may overhear this sensitive information and technology users have no ability to control what kinds of information a voice interface may say aloud (Williams et al., 2022).

#### 3.2.4 Summary of lightning talks

Through their lightning talks, our participants contributed to an initial gathering of different troubles and failures in conversational interactions between humans and robots. Thanks to the description of their memorable failures and their analysis, we could identify the themes of *analysis*, *technical aspects* and *adjacent topics*, which all impact the success (or failure) of a conversation.

### 3.3 Summary of World Café session

During the World Café session, four working groups were created based on recurring themes from the lightning talks, participants’ answers as to what they perceived as the most pressing issue or the biggest source of failure for speech interfaces, as well as the aim to define the sought after benchmark scenario. Through the initial submissions of the participants, their lightning talks and the keynotes, three main macro-categories have emerged: i) miscommunication, ranging from speech recognition failures to more semantic and conversation-dependent failures; ii) interaction problems, encompassing all those failures that are due to users’ expectations and behaviours; iii) context understanding, linked to the fact that interaction is shaped by context and that context changes fast, calling for a need to find more robust ways to establish common ground. While these three themes are highly interdependent and could culminate in the sought after benchmark scenario (the fourth working group), each of them presents peculiarities that we considered worth discussing in detail.

#### 3.3.1 Handling Miscommunication

The discussion focused on the need to acknowledge and embrace the concept of miscommunication. One of the open challenges identified by this group was to equip robots with the ability to learn from various forms of miscommunication and to actively use them as an opportunity to establish common ground between users and robots. When communicating with a robot, the human user usually has a goal in mind. The robot could exploit miscommunication to understand this goal better by asking for clarifications at the right moments and updating the common ground. The discussion also acknowledged that miscommunication is only the starting point. Two distinct new challenges and opportunities arise when working on resolving miscommunication: 1) how to explain the miscommunication, and 2) how to move the conversation forward. Both problems are highly context-dependent and related to the severity and type of miscommunication. Moreover, being able to repair a breakdown in conversation may also depend on being able to establish appropriate user expectations in the first place by giving an accurate account of what the robot is really able to accomplish. The final discussion point from this group centered on the possibility of enriching the multimodal and non-verbal component of conversations to help the robot perceive when a miscommunication has happened by detecting and responding to, for example, long pauses or changes in specific types of facial expressions.

#### 3.3.2 Interaction problems

Interaction problems do not only encompass challenges that are specific to the technology used, like issues with automatic speech recognition or the presence of long delays when trying to engage in a “natural” conversation. They are related to perceived failures that longitudinally include all the technical problems identified by the other themes and relate to how the interaction with the human user is managed. In this context, human users play an essential role and the creating expectations that allow users to build an adequate mental model of the technology they are interacting with. In Washburn et al. (2020a), authors examine how expectations for robot functionality affected participants’ perceptions of the reliability and trust of a robot that makes errors. The hope is that this would lead to an increased willingness and capacity to work with the failures that inevitably occur in conversational interactions. Anthropomorphism was identified as one of the possible causes for the creation of wrong expectations: the way robots both look and speak risks tricking users into thinking that robots have human-like abilities and are able to follow social norms. Once this belief is abandoned, users could then form an appropriate expectation of the artificial agents, and the severity of the failures would decrease. Setting the right expectations will also enable users to understand when a failure is a technological error in execution or when it is a design problem: humans are unpredictable, and some of the problems that arise in the interactions are due to users’ behaviours that were not embedded in the design of robot’s behaviours. A related aspect that was considered important by this group is the transparency of the interaction: the rationale behind the failures should be explained and made clear to the users to enable mutual understanding of the situation and prompt recovery. This could, in fact, be initiated by the users themselves. Another need, identified as a possible way to establish better conversational interactions, is the missing link of personalisation. The more the agents are able to adapt to the context and the users they are interacting with, the more they will be accepted, as acceptance plays a fundamental role in failure management. A general consensus converged regarding the fact that we are not yet at the stage where we can develop all-purpose chatbots - or robots - and the general public should be made aware of this, too. Each deployment of conversational agents is context related and the conversation is mainly task-oriented, where a precise exchange of information needs to happen for a scenario to unfold.

#### 3.3.3 Context understanding

All four groups agreed that context understanding is crucial for reducing or entirely eliminating failures of interactive systems that use spoken language. We determined that capturing and modelling context is particularly challenging since it is an unbound and potentially all-encompassing problem. Moreover, all dialogue, and in fact, interaction as a whole, would be *shaped by* the context while at the same time *renewing* it. Likewise, the volatility of context, in particular, potentially rapid context switches, was also identified as challenging in human-robot conversation. Modelling the interaction partner(s) and evaluating their focus of attention was thereby discussed as one potential approach to reducing context search space.

A precise and consistent representation of the dialogue context was therefore identified as one of the most important problems that would rely on modelling not only the current situation but also any prior experiences of humans with whom the system is interacting. Such previous experience was seen to have significant effects on expectations about the interactive system that would potentially require calibration before or during system runtime to avoid misunderstandings as well as misaligned trust towards the system Hancock et al. (2011). However, even if we assume an optimal representation of context would be possible, the problem of prioritisation and weighting would still persist.

Another challenge discussed was the need for a multi-modal representation of the current situation comprised of nonverbal signals, irregular words, and interjections. Such a model would be required for an appropriate formulation of common ground, whereby it remains unclear what exactly would be required to include. In that context, one group identified the benefits of a typology that could encompass an interaction situation in a multi-modal way, potentially extending work by Holthaus et al. (2023). The exact mapping between a signal or lexical index and their meanings is, however, still difficult to establish.

On the other hand, considering the dialogue context was unanimously regarded as beneficial to enrich human-robot conversations offering numerous opportunities to increase its functionality, even if it would not be possible to capture all context comprehensively. With a personalised model of interaction partners, for example, the spoken dialogue could be enhanced by taking into account personal interaction histories and preferences. Conversational agents could be improved for highly constrained settings and converge faster to relevant topics.

It is noteworthy to mention that enriching the capabilities of conversational agents with context information poses ethical challenges, e.g., in terms of privacy and data protection. This approach might thus introduce barriers in terms of user acceptance that need to be considered Lau et al. (2018). However, using context appropriately could also help to improve a system’s transparency either by designing it with its intended context in mind or by utilising it during a conversation, for example, by providing additional interfaces to transport further information supporting the dialogue or by analysing context to reduce ambiguities and eliminate noise. The context was regarded to often play a vital role in providing the necessary semantic frame to determine the correct meaning of spoken language. Making use of domain and task knowledge was thereby identified as particularly helpful.

Moreover, intentionally misapplying context or analysing situations where context has previously misled a conversation, might be avenues to recognize and generate error patterns to help detect future troubles and failures in speech understanding.

#### 3.3.4 Benchmark Scenario(s)

On this discussion table, participants struggled to devise a single benchmark scenario that would elicit most, if not all, commonly occurring conversational failures. As a main reason for the difficulty of identifying such a prototypical scenario, the lack of a comprehensive taxonomy of conversational failures was determined.

An alternative suggestion to the proposed task of identifying one, failure-wise all encompassing, scenario was also made. Rather than seeking to specify a single scenario, it may be necessary to create test plans for each specific interaction task using chaos engineering, with some of the defining characteristics for a scenario being 1) the type(s) of users, 2) the domain of use (e.g., health-related, shopping mall information kiosk), 3) the concrete task of the robot, 4) the types of errors under investigation. Chaos engineering is typically used to introduce a certain level of resilience to large distributed systems (*cf.* Fomunyam (2020). Using this technique, large online retailers such as Amazon deliberately knock out some of their subsystems, or introduce other kinds of errors, to ensure that the overall service can still be provided despite the failure of one or more of these, typically redundant, components (cf. Siwach et al., 2022). While both the envisioned benchmark scenario(s) and chaos engineering are meant to expose potential failures of human-made systems, the types of systems and types of failure differ substantially. While failures in technical distributed systems are unilateral, in the sense that the source of failure is typically attributed solely to the system rather than its user, attribution of blame in conversational failure is less unilateral. If a successful conversation is seen to be a joint achievement of at least two speakers, conversational failure is probably also best seen as a joint “achievement” of sorts. In other words, the *user* of a conversational robot is always also an interlocutor during the interaction. Hence, whatever approach we use to identify and correct conversational failures, the correct level of analysis is that of the dyad rather than of the robot alone.

Independent of the chaos engineering approach, another suggestion was that at least two benchmarks might be needed in order to distinguish between low-risk and high-risk conversations. Here, low-risk conversations would be the more casual conversations that one may have with a shop assistant whose failure would not carry any hefty consequences. High-risk conversations, on the other hand, would be those where the consequences of conversational failure might be grave - imagine conversational failure between an assistive robot and its human user that are engaged in some joint task of removing radioactive materials from a decommissioned nuclear site. If such a distinction should be made, the logical follow-up question would be how the boundary between low and high-risk scenarios should be determined. Finally, it should be mentioned that at least partial benchmarks such as *Paradise* exist for the evaluation of spoken dialogue systems Walker et al. (1997).

## 4 Discussion

One significant result from the workshop is that no succinct and, more importantly, singular benchmark scenario could be envisioned that would likely elicit all or, at least, a majority of identified failures. A likely reason behind this is the lack of a comprehensive categorization of conversational failures and their triggers in mixed human-machine interactions. Having such a taxonomy would allow us to embed such triggers systematically in benchmark scenarios.

### 4.1 Wanted: A taxonomy of conversational failures in HRI

Honig and Oron-Gilad (2018) recently proposed a taxonomy for failures in HRI based on a literature review of prior failure-related HRI studies. Their survey indicated a great asymmetry in these investigations, in that the majority of previous work focused on technical failures of the robot. In contrast, Honig & Oron-Gilad noticed that no strategies had been proposed to deal with “human errors”. From a conversation analytic viewpoint, the dichotomy of technical vs human error may not always be as absolute when applied to conversational failures, especially since, despite sharing some terminology, CA conceptualizes conversational success and failure quite differently. Conversation analysts conceive of successful conversation as the achievement of joint action by any party (robot or human). In this sense, when a failure occurs, the “blame” lies with all participants. Similarly, success in CA terms might mean that a joint action is “successfully” achieved interactionally, even if there are informational errors. For example, an invitation to meet under the clock at Grand Central station, where the recipient misunderstands the time/place might be “successfully” achieved as an orderly interaction, the error being marked. In HRI, however, this failure of the “Schelling game” would be considered a classic “grounding error” Clark (1996), and it would certainly matter who made the error: the human or robot. While not assigning blame for some singular failure simultaneously to both participants, Uchida et al. (2019a) recently used a blame assignment strategy where the responsibility for a sequence of failures was attributed in an alternating fashion to the robot and the human. As indicated by our struggle to find a good general characterisation of conversational failures during the workshop, we advocate the construction of a taxonomy of conversational failures for mixed, that is human-machine dyads and groups. To build such a taxonomy, an interdisciplinary effort is needed, given that the types of relevant failures span the entire spectrum from the very technical (e.g., ASR errors) to the very “relational” (e.g., misunderstanding based on lack of common ground). The relevant disciplines would include linguistics, conversation analysis, robotics, NLP, HRI, and HCI. This workshop represented the first stepping stone towards this interdisciplinary effort. One theory-related advantage of taxonomy building is that it forces us to reconsider theoretical constructs from different disciplines, thereby potentially exposing gaps in the respective theories - similarly to how conversation analysis has exposed shortcomings of speech act theory (*cf.* Levinson, (1983)).

The process of defining the types of errors could also help us to understand why they arise, measure their impact and explore possibilities and appropriate ways to detect, mitigate and recover from them. If, for example, artificial agents and human users are mismatched conversational partners as suggested by Moore (2007) and Förster et al. (2019), and if this mismatch creates constraints and a “habitability gap” in HRI (Moore, 2017), are their specific types of failures that only occur due to such asymmetric setups? And, if yes, what does that mean for potential error management in HRI? If priors shared between interlocutors matter (Huang and Moore, 2022; Moore, 2022), how does the aligning of interactive affordances help to increase the system’s capacity to deal with errors? Moreover, errors can affect people’s perception of a robot’s trustworthiness and reliability (e.g., Washburn et al., 2020b), as well as their acceptance and willingness to cooperate in HRI (e.g., Salem et al., 2015). What type of errors matters more? In terms of error recovery, it has been shown that social signals, such as facial action unit (AU), can enhance error detection (Stiber et al., 2023); Users’ cooperative intention can be elicited to avoid or repair from dialogue breakdowns (Uchida et al., 2019b). The question is, when facing different errors, do these strategies need to be adaptable to tasks/scenarios, and if so, to what degree? Answering the above questions requires a deeper understanding of conversational failures, and taxonomy building is one possible way to increase our understanding.

A more practical advantage of having such a taxonomy is discussed in the next section.

### 4.2 Benchmarking multimodal speech interfaces

One of the intended aims of the workshop was to define, or at least outline, some benchmark scenario that would have the “built-in” capacity to expose, if not all, at least a good number of potential communicative failures of some given speech interface. During the workshop, it became apparent that we would fail to come up with such a single scenario. It questionable whether such a scenario could exist or whether a number of scenarios would be needed to target different settings in which the speech interface is to be deployed. One main reason for our struggle that emerged during the World Café session was the lack of a taxonomy of communicative failures in HRI. Having such a taxonomy would allow the designer, or user, of a speech interface to systematically check whether it could handle the type of situation in which the identified failures are likely to occur prior to testing it “in the wild”.

Related to the construction of a potential (set of) benchmarks is the question of how to evaluate multimodal speech interfaces. The popular evaluation framework PARADISE Walker et al. (1997), originally designed for the assessment of unimodal dialogue systems, has already been used in multimodal HRI studies (e.g., Peltason et al., 2012; Giuliani et al., 2013; Hwang et al., 2020). Also within the HCI community multimodal alternatives to PARADISE have been proposed (e.g., Kühnel, 2012). Given these existing evaluation frameworks for multimodal dialogue systems, what would a failure-based method bring to the table?

A characteristic of PARADISE and related frameworks is that they tend to evaluate a past dialogue according to a set of positive performance criteria. PARADISE, for example, uses measurements of *task success*, *dialogue efficiency*, and *dialogue quality* to score a given dialogue. There is likely an inverse relationship between a failure-based evaluation and, for example, *dialogue efficiency* as a dialogue containing more failures, will likely require more turns to accomplish the same task due to repair-related turns. This would mean that the efficiency of this failure-laden dialogue would be reduced. However, despite this relationship, the two methods are not commensurate. A failure-based scoring method could, for example, put positive value on the resilience of some speech interface, by assigning positive values to the number of successful repairs. This would, in some sense, be diametrically juxtaposed to efficiency measures. On the other hand, these two ways of assessing a speech interface are not mutually exclusive and could be applied simultaneously.

One interesting observation with respect to the surveyed studies points to a potential limitation of existing evaluation frameworks such as PARADISE. All of the referenced studies are based on turn-based interaction formats. While turn-based interaction is certainly a common format in many forms of human-human and human-robot interaction, it is likely not the only one. Physical human-robot collaboration tasks which require participants to coordinate their actions in a near-simultaneous manner, for example, when carrying some heavy object together, do not necessarily follow a turn-based format. While some of the involved communication channels such as speech will likely be turn-based, other channels such as sensorimotor communication (SMC, *cf.* Pezzulo et al., 2019) may or may not follow this format.

## 5 Conclusion

The first workshop on “Working with Troubles and Failures in Conversation between Humans and Robots” was the first effort to gather an interdisciplinary team of researchers interested in openly discuss the challenges and opportunities in designing and deploying speech interfaces for robots. Thanks to insights from conversation analysis, cognitive science, linguistics, robotics, human-robot interaction, and dialogue systems, we initiated a discussion that does not simply dismiss failures in conversational interaction as a negative outcome of the robotic system, but engages with the nature of such failures and the opportunities that arise from using them to improve the interactions. We believe this initial push will spawn a deeper research effort towards the identification of a benchmark for multimodal speech interfaces and the creation of a systematic taxonomy of failures in conversation between humans and robots which could be useful to interaction designers, both in robotics and non-robotics fields.

## Figures and Tables

**Table:** 

**Voice interfaces**	User interfaces that allow interaction with technology through spoken commands or queries
**Robotic speech interfaces**	Voice interfaces applied on robots that use both speech recognition as well as synthesised or artificial voices to communicate and interact with users
**Chatbots**	Text-based interfaces able to provide information, answer questions, or assist with various tasks
**Agents, artificial agents, conversational agents**	Terms used interchangeably for systems designed to engage in natural language conversations with humans, by employing natural language processing and machine learning to understand and respond to user queries, provide information or assistance

## Data Availability

The original contributions presented in the study are included in the article/Supplementary Material, further inquiries can be directed to the corresponding author.

## Footnotes

1
https://alanaai.com/

2
https://theworldcafe.com/key-concepts-resources/world-cafe-method/

3
https://peppermint.projet.liris.cnrs.fr/

4
https://www.canopies-project.eu/
